# Notices of Bills and Returns, and of the Report of Mr. Simon on Cholera

**Published:** 1856-07

**Authors:** 


### SANITARY LEGISLATION.

Our report on sanitary legislation must of necessity be very brief; and, indeed, there is not much to communicate.

BILLS.

*The Nuisances Removal Bill for Scotland* has been revised, and so remains.

*A Bill to amend the Public Health Act of* 1848 has been brought in by Mr. Cowper and Sir George Grey, and printed. Its provisions are too important to be treated of here cursorily.

*A Bill,* intitled an *Act to amend the Smoke Nuisance Abatement* (*Metropolis*) *Act,* brought from the Upper House, provides that certain glass-works and furnaces in the metropolis, and steam-vessels above London Bridge, shall, after Jan. 1, 1858, be no longer exempt from the operation of the said act.

*A Bill to Amend the Burial Acts,* prepared by Mr. Massey and Sir G. Grey, suggests that the General Board of Health shall be substituted for the Secretary of State, for the purposes of burial acts; that burial boards may provide more than one burial ground; that local boards of health may, by order in Council, be constituted burial boards.

RETURNS.

A Return from the General Board of Health, shows that the Public Health Act has been applied during the years 1854-55 to the following towns:—Plymouth; Haworth, Yorkshire; Aberdare, Glamorganshire; Bishop Auckland, Durham; Willenhall, Staffordshire; Over Darwen, Lancashire; Crumpsall, Lancashire; Arnold, Notts; Heanor, Derbyshire; Wanstead, Essex; Garston, Lancashire; Houghton-le-Spring, Durham; Barton-upon-Irwell, Lancashire; Burley, Yorkshire; Malton, Yorkshire; Middlesbrough, Yorkshire; Windhill, Yorkshire; Christchurch, Monmouthshire; Keighley, Yorkshire; Tunstall, Staffordshire; Toxteth Park, Lancashire; Romford, Essex; Wilton, Wiltshire; Eccleshill, Yorkshire; Tipton, Staffordshire; Mistley, Essex; Redcar, Yorkshire; Wellingborough, Northamptonshire; and Smethwick, Staffordshire, in 1856. Portions of the act have also been incorporated in local acts in the following places:—Bolton, Lancashire; Burnley, Lancashire; Kingston-upon-Hull; Ryde, Isle of Wight; Wellington, Salop; and Shrewsbury.

REPORTS.

A Report on the Cholera Epidemics of London, as affected by the consumption of impure water, has been brought out by Mr. Simon. We are surprised to find one deep blot in this report; but whether it be a sin of omission or commission we will not pretend to say. We hope the former. Here is the complaint. The report, as far as it goes, tends conclusively to establish the correctness of those views on the propagation of cholera, which Dr. Snow was the first to originate, to announce, and in the midst of violent opposition, amounting even to ridicule, to prove by a series of statistical and personal inquiries, carried on by his own unaided industry and at immense pecuniary cost. To suppose that Mr. Simon was not acquainted with the labours of Dr. Snow is absurd; yet in the report he has not once referred to that gentleman, nor acknowledged in an admitted discovery the worth of the discoverer. We hope Dr. Snow is careless to such slights, intentional or accidental. He may well be so. He has only to bide his time. His claims to originality are clear as noonday; and when in posterity the full influence of his labours are felt, his name will be accepted, as of one, who by genius for observation has conferred a signal and lasting blessing on his fellow-men. Then the slights of this poor, proud, paltry, selfish age, will be spoken of only as to his honour and to the truth of his mission.

